# Digital Health: Improve Patient Care while Preserving the Planet, an Ethical Imperative: The paradox of innovation between clinical benefits and ecological limits

**DOI:** 10.1055/s-0046-1816586

**Published:** 2026-11-03

**Authors:** Brigitte Séroussi

**Affiliations:** 1 Sorbonne Université, AP-HP, Tenon Hospital, Public Health Department, Inserm, Université Sorbonne Paris Nord, Limics75006ParisFrance

## 1. Introduction

Digital health promises better, faster, and more preventive care. Yet this revolution is not an unlimited resource: every algorithm, server, and device consumes energy, raw materials, and water, contributing to greenhouse gas emissions and environmental stress. How can we advance patient care without undermining the very conditions that sustain life on Earth? At COP26 in Glasgow, and reaffirmed at COP28 in Dubai, nearly 50 countries committed to building resilient, low-carbon health systems. These pledges marked a first step toward a unified global response to both healthcare vulnerabilities and their environmental impacts. But if digital health is to fulfill its promise, it must embrace sobriety, deploying innovation only when the patient benefit outweighs its ecological footprint.

## 2. The Climate Challenge and Health Systems

The World Health Organization identifies health system resilience as a top priority, with sustainability as its foundation: care that continues without exhausting financial, ecological, or social resources. Climate change, however, remains the greatest health threat of the 21st century. The Intergovernmental Panel on Climate Change (IPCC) warns of a possible 4°C global temperature rise by 2100—or at least 2°C by 2050 even under strong mitigation. The consequences—food insecurity, pollution, emerging diseases, and mental health challenges—already disproportionately affect the most vulnerable populations.

Ironically, healthcare itself contributes significantly to this problem. The sector accounts for 4–12% of national greenhouse gas emissions, depending on the country. Digital health, often perceived as “immaterial,” is in reality deeply material: data centers, networks, and devices demand energy, water, and raw materials. Globally, digital technologies now account for roughly 4% of greenhouse gas emissions—a share comparable to commercial aviation—and healthcare’s contribution continues to grow.

## Two Lenses: Green4IT and IT4Green

To navigate this challenge, we can adopt two complementary perspectives: Green4IT is making digital itself more sustainable, and IT4Green covers using digital to reduce healthcare’s overall environmental footprint. Both are necessary if innovation is to respect planetary boundaries.

Green4IT or Reducing Digital Health’s Footprint: A life-cycle analysis of digital health reveals its hidden costs: manufacturing, transport, usage, and disposal. Historically, manufacturing dominated, representing roughly 75% of the footprint. Extending device lifespans, promoting refurbishment and recycling, and favoring durable, repairable equipment were thus the most effective levers. Governments are stepping in. In France, the Ministry of Health now provides “ecoscores” for software developers—metrics measuring energy use, data flows, and performance. Hospitals experiment with deleting obsolete data, reusing server waste heat, and donating refurbished devices. Individually small, these steps collectively signal a shift toward responsibility. Yet the balance is changing. With the rise of Artificial Intelligence, usage now rivals manufacturing in environmental impact. Massive model training consumes enormous energy and water for cooling, turning what was once a 75/25 split into 40/60 or even higher. The lesson: digital health cannot be an “open bar.” Just as clinicians avoid overdiagnosis, informaticians must avoid over-computation. **Digital sobriety** —using technology only when necessary—is now a practical imperative.

IT4Green or Using Digital to Support Sustainability: Digital technologies can also be powerful tools for reducing healthcare’s footprint. Teleconsultations limit travels, electronic records prevent redundant tests, remote monitoring can reduce admissions, and predictive analytics guide prevention. But benefits are not automatic: telehealth that supplements rather than replaces in-person visits can increase emissions, and remote monitoring that generates low-value data can consume energy without clinical gain. True IT4Green requires discernment, proportionality, and rigorous evaluation of ecological and clinical outcomes.

## 4. The Paradox of Digital Health

Here lies the core paradox: digital health is promoted as a driver of patient benefits—improving diagnosis, accessibility, continuity, and prevention—yet its deployment must be restrained due to its environmental burden. How can a sector devoted to human care contribute so heavily to harming the very conditions of human life? How do we reconcile the promise of digital transformation with planetary boundaries?

No innovation is immaterial. Every byte, algorithm, and device has a footprint. The question is not whether digital health impacts the environment, but **how we manage it** .

Four priorities emerge:

- **Measure** : ecoscores, carbon calculators, and life-cycle analyses must become routine;
- **Educate** : train engineers in eco-design, clinicians in digital sobriety, and managers in sustainable procurement;
- **Incentivize** : Procurement frameworks and funding should reward eco-responsibility;
- **Reframe value** : Environmental impact must sit alongside safety, efficacy, and equity as a criterion of digital health quality.

## 5. A Broader Horizon

Ultimately, digital health challenges us to define what progress truly means. Technology is never neutral—it reflects choices and priorities. Unlimited digital growth ignores planetary limits. Responsible digital health—rooted in eco-design, sobriety, and essential use—acknowledges these limits. Digital health promised to heal. Now it must prove it can do so without exhausting the planet’s resources. The challenge ahead is not only technical but ethical: ensuring the healing arts do not harm the Earth. As biomedical informatics experts, we are uniquely positioned to lead this reflection. We design systems, set standards, and shape the culture of digital health. Our responsibility is not only to innovate, but to innovate consciously—measuring impacts, questioning necessity, and aligning progress with sustainability.

